# Functional consequence of myeloid ferritin heavy chain on acute and chronic effects of rhabdomyolysis-induced kidney injury

**DOI:** 10.3389/fmed.2022.894521

**Published:** 2022-09-08

**Authors:** Kayla R. McCullough, Juheb Akhter, Mauhaun J. Taheri, Amie Traylor, Anna A. Zmijewska, Vivek Verma, Matthew C. Hudson, Abhishek Sachdeva, Elise N. Erman, Kyle H. Moore, James F. George, Subhashini Bolisetty

**Affiliations:** ^1^Department of Medicine, University of Alabama at Birmingham, Birmingham, AL, United States; ^2^Nephrology Research and Training Center, University of Alabama at Birmingham, Birmingham, AL, United States; ^3^Department of Surgery, University of Alabama at Birmingham, Birmingham, AL, United States; ^4^Department of Cell, Developmental and Integrative Biology, University of Alabama at Birmingham, Birmingham, AL, United States

**Keywords:** ferritin, inflammatory response, rhabdomyolysis, iron, macrophages, kidney, fibrosis, ferritin heavy chain

## Abstract

Acute kidney injury (AKI) is a serious complication of rhabdomyolysis that significantly impacts survival. Myoglobin released from the damaged muscle accumulates in the kidney, causing heme iron-mediated oxidative stress, tubular cell death, and inflammation. In response to injury, myeloid cells, specifically neutrophils and macrophages, infiltrate the kidneys, and mediate response to injury. Ferritin, comprised of ferritin light chain and ferritin heavy chain (FtH), is vital for intracellular iron handling. Given the dominant role of macrophages and heme-iron burden in the pathogenesis of rhabdomyolysis, we studied the functional role of myeloid FtH in rhabdomyolysis-induced AKI and subsequent fibrosis. Using two models of rhabdomyolysis induced AKI, we found that during the acute phase, myeloid FtH deletion did not impact rhabdomyolysis-induced kidney injury, cell death or cell proliferation, suggesting that tubular heme burden is the dominant injury mechanism. We also determined that, while the kidney architecture was markedly improved after 28 days, tubular casts persisted in the kidneys, suggesting sustained damage or incomplete recovery. We further showed that rhabdomyolysis resulted in an abundance of disparate intra-renal immune cell populations, such that myeloid populations dominated during the acute phase and lymphoid populations dominated in the chronic phase. Fibrotic remodeling was induced in both genotypes at 7 days post-injury but continued to progress only in wild-type mice. This was accompanied by an increase in expression of pro-fibrogenic and immunomodulatory proteins, such as transforming growth factor-β, S100A8, and tumor necrosis factor-α. Taken together, we found that while the initial injury response to heme burden was similar, myeloid FtH deficiency was associated with lesser interstitial fibrosis. Future studies are warranted to determine whether this differential fibrotic remodeling will render these animals more susceptible to a second AKI insult or progress to chronic kidney disease at an accelerated pace.

## Introduction

Rhabdomyolysis is caused by the breakdown of muscle, leading to the release of intracellular content into systemic circulation. A common feature during rhabdomyolysis is the development of acute kidney injury (AKI). This association was documented during World War II by Bywaters and Beall who noticed that victims of crush injury had dark urine and reduced urinary output. It is now clear that there are multiple causes of rhabdomyolysis including strenuous exercise in a volume depleted state, drugs, insect bites, infections, and others. Notably, it is estimated that nearly one-tenth of patients with rhabdomyolysis experience AKI. Importantly, AKI significantly impacts morbidity and mortality in these patients, and in survivors, may further impact the progression to chronic kidney disease (CKD) (1–4).

During rhabdomyolysis, myoglobin released from the muscle accumulates in the kidney, causing damage *via* direct and indirect mechanisms. These include heme-mediated oxidative stress and cell death in the proximal tubules, endothelial dysfunction, vasoconstriction, increased cast formation, and inflammation (4–6). In this context, we and others have documented the salutary role of antioxidants and anti-inflammatory mediators in protection against AKI (7–14). Also, AKI severity is dependent on the proximal tubular expression of heme oxygenase, an enzyme that catalyzes the breakdown of heme into equimolar quantities of ferrous iron, carbon monoxide and biliverdin (11–13, 15). Iron released from this reaction can potentiate oxidative stress through generation of reactive oxygen species *via* the Fenton reaction. However, this is largely inhibited by rapid upregulation of ferritin, which sequesters and stores iron. Ferritin exists as a shell comprised of heavy and light chain subunits. Ferritin heavy chain (FtH) is a ferroxidase which oxidizes the ferrous iron to the ferric form, and ferritin light chain (FtL) enables ferric iron nucleation within the ferritin shell. In recent work, we demonstrated that proximal tubular FtH deletion exacerbates AKI and leads to significant mortality during rhabdomyolysis (16). These studies were corroborated by the use of iron chelators and inducers of heme oxygenase expression within the kidney (10, 17–20).

Experimental evidence also indicates that during rhabdomyolysis, immune cells, specifically neutrophils and macrophages, accumulate in the kidney and mediate injury and/or repair. Specifically, depletion of macrophages using liposomal clodronate prior to induction of rhabdomyolysis or during the initial injury phase was protective (21, 22). Another elegant study recently demonstrated that macrophages release extracellular traps comprised of DNA fibers and granule proteins that mediate kidney injury during rhabdomyolysis (23). Macrophages also participate in kidney fibrosis and repair. These contrasting functions of macrophages are thought to be mediated by phenotypically distinct subpopulations of macrophages that bidirectionally communicate with the kidney parenchyma (24–30).

It is becoming increasingly evident that AKI is a risk factor for the development of CKD. Severity of AKI and repeated insults to the kidney correlate with an increased rate of progression to CKD (31–34). Given the dominant role of macrophages and dysregulated iron metabolism in the pathogenesis of rhabdomyolysis, we designed this study to evaluate the functional role of macrophage (myeloid) ferritin in rhabdomyolysis-induced AKI and subsequent fibrosis.

## Materials and methods

### Experimental model

Ferritin heavy chain floxed mice (FtH^fl/fl^) and myeloid-specific ferritin heavy chain knockout mice (FtH^LysM–/–^) were used for this study and have been previously described (35). Briefly, myeloid-specific FtH knockout mice, on a C57BL/6 background were generated by breeding FtH^fl/fl^ and LysM Cre mice. All mice were humanely treated, and all methods were approved by the Institution Animal Care and Use Committee of the University of Alabama at Birmingham (Approval – 21255). Studies were executed in accordance with all National Institutes of Health guidelines.

### Glycerol-induced rhabdomyolysis

Two separate protocols were used to induce rhabdomyolysis: (1) single dose of glycerol administration, (2) unilateral nephrectomy followed by glycerol administration. Glycerol mediated rhabdomyolysis was induced in age matched FtH^fl/fl^ and FtH^LysM–/–^ mice as previously described (13). Briefly, mice were deprived of water for 16 h prior to glycerol administration. Mice were anesthetized with isoflurane and injected with 50% glycerol in water, 7.5 ml/kg body weight, with half the volume delivered into each anterior thigh muscle. A cohort of mice underwent unilateral nephrectomy and 2 weeks later, the mice were deprived of water and administered glycerol as described above. Mice were sacrificed at various timepoints following injection and kidneys were analyzed for gene and protein expression. In an additional cohort of mice, blood was collected *via* facial vein from the same animals at multiple time-points. Blood was collected *via* cardiac puncture or facial vein bleed, and serum was isolated for measurement of creatinine and other injury markers. Urine was collected at the time of harvest *via* bladder puncture and analyzed for markers of kidney injury.

### Serum and urine analysis

ELISA analyses for KIM-1 (Cat no. MKM100, R&D Systems), NGAL (Cat no. MLCN20, R&D Systems), Cystatin C (Cat no. MSCTC0, R&D Systems), and Albumin (Cat no. E99-134, Bethyl Laboratories) were performed on serum or urine following the manufacturer’s instructions. Urine and serum creatinine were measured by LC-MS/MS. Data were expressed as nanograms per milliliter or were normalized to urine creatinine and expressed as nanograms per milligram creatinine.

### Quantification of mRNA expression

Total RNA was isolated from tissues using TRIzol reagent (Invitrogen), cDNA was generated from the total RNA using the Maxima H Minus First Strand cDNA Synthesis Kit (Cat no. K1651, Thermo Scientific). SYBR Green-based real-time PCR was performed on the cDNA using PowerUp SYBR Green Master Mix (Cat no. A25742, Applied Biosystems). Relative mRNA expression was quantified using the ΔΔCt method. All values were normalized to GAPDH as an internal control. All reactions were performed in triplicate and melting curve analysis was used to confirm specificity. All real-time primers used are listed in Table 1.

**TABLE 1:** Primers for real-time PCR analysis.

Gene	Primer sequence (5′-3′)
GAPDH Fwd	ATCATCCCTGCATCCACT
GAPDH Rev	ATCCACGACGGACACATT
H-Ferritin Fwd	CCATCAACCGCCAGATCAAC
H-Ferritin Rev	GAAACATCATCTCGGTCAAA
KIM-1 Fwd	AGGAAGACCCACGGCTATTT
KIM-1 Rev	TGTCACAGTGCCATTCCAGT
Fibronectin Fwd	TGTCACAGTGCCATTCCAGT
Fibronectin Rev	CACTGAAGCAGGTTTCCTCGGTTGT
TNF-α Fwd	ACGGCATGGATCTCAAAGAC
TNF-α Rev	AGATAGCAAATCGGCTGACG
NGAL Fwd	CACTGAAGCAGGTTTCCTCGGTTGT
NGAL Rev	CACTGAAGCAGGTTTCCTCGGTTGT
Col1 Fwd	GCTCCTCTTAGGGGCCACT
Col1 Rev	CCACGTCTCACCATTGGGG
α-SMA Fwd	CACTGAAGCAGGTTTCCTCGGTTGT
α-SMA Rev	CACTGAAGCAGGTTTCCTCGGTTGT
TGF-β Fwd	CTCCCGTGGCTTCTAGTGC
TGF-β Rev	GCCTTAGTTTGGACAGGATCTG

### Western blot analysis

Harvested tissues were homogenized and protein was isolated as previously described (35). Briefly, tissues were lysed in protein lysis buffer (10 mM/1 Tris–HCl, 5 mM/1 EDTA, 150 mM/1 NaCl, 10% Non-idet P-40, and 10% Triton-X) with protease (Sigma-Aldrich) and phosphatase (Biotool) inhibitors. Protein was isolated and quantified with the bicinchoninic acid protein assay (Cat no. 23227, ThermoFisher). Protein (50 μg) was resolved on 12% Tris-glycine sodium dodecyl sulfate polyacrylamide gel electrophoresis or 4–12%, BIS-TRIS polyacrylamide gel (ThermoFisher) and transferred to a PVDF membrane (EMB Millipore). Membranes were blocked with 5% non-fat dry milk in TBST for 1 h and then incubated with the following antibodies: a mouse anti-FtH (Cat no. sc517348, Santa Cruz, 1:1,000), a rabbit anti-phospho-JNK (Cat no. 4668, Cell Signaling Technology, 1:1,000), a rabbit anti-JNK (Cat no. 9252, Cell Signaling Technology, 1:1,000), a rabbit anti-phospho-p44/42 MAPK (Cat no. 9101, Cell Signaling Technology, 1:1,000), a rabbit anti-p44/42 MAPK (Cat no. 4695, Cell Signaling Technology, 1:1,000), a rabbit anti-fibronectin (Cat no. F3648, Sigma-Aldrich, 1:10,000), a mouse anti-smooth muscle actin (Cat no. A6228, Sigma-Aldrich, 1:5,000), a mouse anti-4-HNE (Cat no. MA33249, R&D Systems, 1:1,000), a goat-anti NGAL (Cat no. AF1857, R&D Systems, 1:2,000), a goat-anti KIM-1 (Cat no. AF1817, R&D Systems; 1:1,000), a mouse anti-GAPDH (Cat no. MAB314, Millipore, 1:10,000) followed by an incubation with peroxidase-conjugated anti-mouse (Kindle BioSciences, Greenwich, CT, United States), or anti-rabbit (Kindle BioSciences), or donkey anti-goat (Cat no. R1002, Kindle BioSciences). Horseradish peroxidase activity was detected using the enhanced chemiluminescence KwikQuant detection system (Kindle BioSciences). Membranes were stripped and reprobed with a mouse anti-GAPDH (Cat no. MAB314, Millipore, 1:10,000) to confirm loading and transfer. Densitometry analysis was performed using ImageStudioLite.

### Immunohistochemistry?

Transverse kidney sections were fixed in 10% neutral buffered formalin for 24 h then embedded in paraffin. Tissues sections were deparaffinized with xylene and rehydrated before antigen retrieval using citrate buffer. Tissues were blocked in normal horse serum, followed by incubation with Ki67 (Cat no. 16667, ABCAM) or 4-HNE (Cat no. MA33249, R&D Systems) or NGAL (Cat no. AF1857, R&D Systems). Sections were washed and then incubated with anti-rabbit (or anti-goat or anti-mouse) secondary antibody (Vector Laboratories) following the manufacturer’s instructions. Sections were washed again and exposed to peroxidase substrate, 3,3-diaminobenzidine (DAB) (Vector Labs), washed in distilled water, dehydrated, mounted, and then visualized by light microscopy. Images were analyzed using ImageJ software (National Institutes of Health). The ApopTag Plus Peroxidase *In Situ* Apoptosis Kit (Cat no. S7101, Sigma-Aldrich) was also used to perform a terminal deoxynucleotidyl dUTP nick end labeling (TUNEL) stain on kidney sections following the manufacturer’s protocol.

### Picrosirius red staining

Collagen deposition was measured using picrosirius red staining as previously described (36). Briefly, tissues were fixed in 10% neutral buffered formalin for 24 h then embedded in paraffin. Tissues were sectioned and deparaffinized with xylene and ethanol washes. The sections were then incubated in picrosirius red for 1 h, washed with acidified water, dehydrated, mounted, and then visualized by light microscopy. Collagen deposition was imaged under polarized light on a Leica DMi8 microscope, and percent area was quantified using Leica LASX software.

### Flow cytometry

The capsules were removed from the harvested kidneys before weighing each kidney. Kidneys were then minced and enzymatically digested using 2 ml 1.67 Wunsch Units per ml Liberase DL (Roche) in DMEM medium (Gibco) for 30 min at 37°C. Cold PEB (PBS, 2 mM EDTA, 1% BSA) was added, and the suspensions were passed through an 18 g needle three times before being filtered through a 40 μm filter (Fisher Scientific). Red blood cells were lysed using the ACK lysis buffer. Samples were incubated with Live/Dead Fixable Violet Dead Cell Stain Kit (Invitrogen) according to the manufacturer’s protocol. After washing with staining buffer (PBS, 0.5% BSA, 0.01% Sodium Azide), samples were incubated in anti-mouse CD16/32 for 10 min prior to incubation with specific primary antibodies. Cells were stained with the following panel for 30 min in the dark at 4°C: Brilliant Violet 650 anti-CD45.2 (BioLegend, clone 104), Super Bright 600 anti-CD11b (eBioscience, clone M1/70), PerCp anti-CD8a (BD Biosciences, clone 53-6.7), FITC anti-CD4 (eBioscience, clone GK1.5), Super Bright 436 anti-Ly6C (eBioscience, clone HK1.4), Alexa Fluor 700 anti-Ly6G (BioLegend, clone 1A8), APC-eFluor 780 anti-F4/80 (eBioscience, clone BM8). Samples were washed in staining buffer, centrifuged at 400 g for 5 min, and the supernatants were aspirated. Samples were then fixed and permeabilized with Foxp3 Transcription Factor Staining Buffer (eBioscience, clone FJK-16s) following the manufacturer’s protocol and stained with APC anti-FoxP3, washed and resuspended in 500 μl of staining buffer for flow cytometry. Data were acquired using a Cytek Northern Lights spectral cytometer with associated SpectroFlo software.

### Statistics

Data are presented as mean ± SEM. For comparisons involving three or more groups, the ANOVA test was used. For repeated measures on the same animal at multiple timepoints, ANOVA test (post-test Dunnett’s, Tukey or Šídák multiple comparison test) was used. Unpaired two-tailed Student’s *t*-test was used for comparison between two groups. A *p*-value of less than 0.05 was considered significant.

## Results

### Myeloid ferritin heavy chain deficiency does not impact rhabdomyolysis induced kidney injury

**FIGURE 1:**
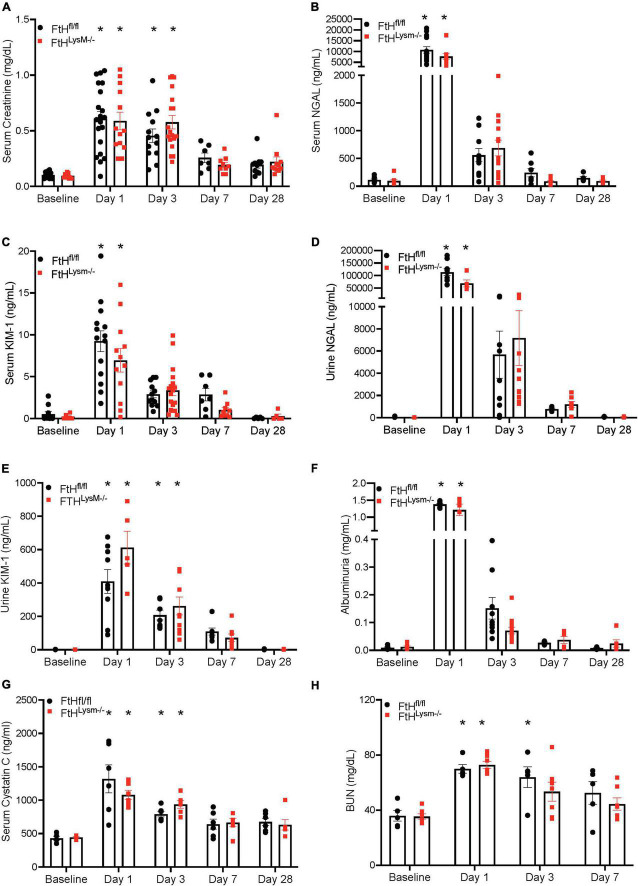
Myeloid FtH deficiency does not impact rhabdomyolysis-acute kidney injury. Mice were administered 7.5 ml/kg body weight of 50% glycerol in water and harvested after 1, 3, 7, or 28 days. Serum was analyzed for **(A)** creatinine expressed in milligrams per deciliter (mg/dl), **(B)** neutrophil gelatinase associated lipocalin (NGAL) expressed in nanograms per milliliter (ng/ml), **(C)** kidney injury molecule-1 (KIM-1) expressed in ng/ml. Urine was analyzed for **(D)** neutrophil gelatinase associated lipocalin (NGAL) expressed in ng/ml, **(E)** kidney injury molecule-1 (KIM-1) expressed in ng/ml, **(F)** albumin expressed in milligrams per milliliter (mg/ml), **(G)** serum cystatin C expressed in ng/ml. **(H)** BUN expressed in mg/dl. All data are expressed as mean ± SEM. *p < 0.05 vs. baseline.

**FIGURE 2:**
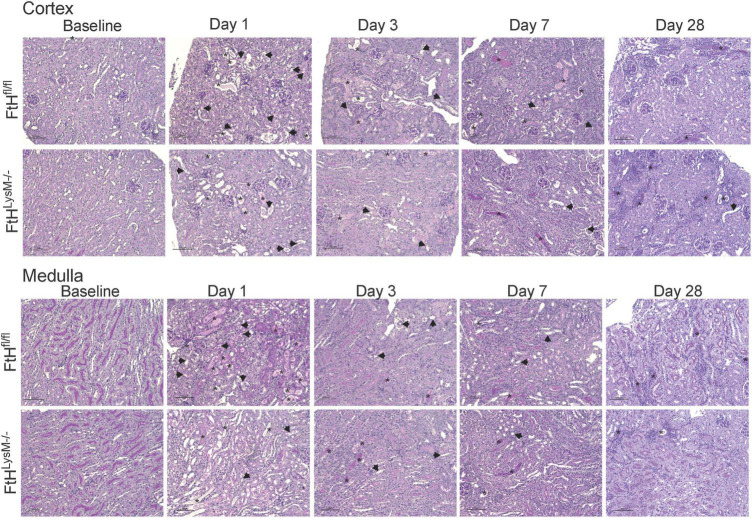
Temporal changes in tubulointerstitial damage following rhabdomyolysis. Representative micrographs demonstrating tubulointerstitial damage in the cortex (upper panel) and outer medulla (lower panel) of kidneys from FtH^fl/fl^ and FtH^LysM–/–^ mice after glycerol administration. Asterisk indicates tubular casts and arrowheads indicate tubular epithelial cell necrosis. Transverse kidney sections were stained with periodic acid–Schiff. *n* = 4–6 per group. The scale bar represents 100 μm.

Given the significance of FtH in the safe sequestration of the potentially detrimental iron, we hypothesized that heme-mediated AKI would be exacerbated in the absence of myeloid FtH. To this end, we administered glycerol to wild-type (FtH^fl/fl^) and myeloid FtH deficient mice (FtH^LysM–/–^) and found similar kidney injury in each genotype during the acute phase (1- and 3-days post-glycerol injection), as evident by a significant rise in serum creatinine (Figure 1A). Similar results were observed in kidney injury markers measured in the serum (NGAL, KIM-1) and urine (NGAL, KIM-1, albumin) at various timepoints following glycerol injection (Figures 1B–H). Of note, repeated blood collections from an additional cohort of mice at various time-points following injury showed an increase in BUN, cystatin C, and creatinine during the acute phase. However, there were no significant differences between the genotypes (Figures 1G,H and Supplementary Figure 1). Renal histology further corroborated these findings with comparable structural damage between the genotypes following injury (Figure 2). An increase in necrotic tubules and tubular casts were identified in the kidney cortex and medulla starting at 1-day post-glycerol injection and persisted until day 7. Interestingly, although fewer in number, casts were present even 28 days post-glycerol administration in both groups.

### Rhabdomyolysis-associated intra-renal signaling mechanisms are not impaired by ferritin heavy chain deficiency

**FIGURE 3:**
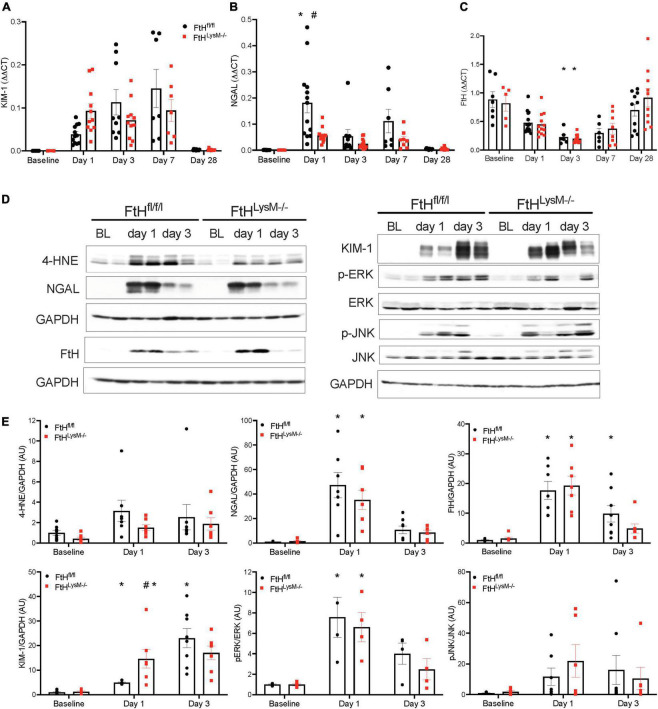
Elucidation of kidney injury markers and MAPK signaling following rhabdomyolysis. Mice were administered 7.5 ml/kg body weight of 50% glycerol in water and harvested after 1 or 3 days. Kidneys were analyzed for the expression of **(A)** kidney injury molecule-1 (KIM-1), **(B)** neutrophil gelatinase associated lipocalin (NGAL), and **(C)** ferritin heavy chain (FtH) by real-time PCR analysis. Each experiment was performed at least three independent times. Results were normalized to GAPDH and presented as mean ± SEM. *p < 0.05 vs. baseline control; ^#^*p* < 0.05 vs. FtH^fl/fl^. **(D)** Kidneys were analyzed for the expression of 4-hydroxynonenal, NGAL, KIM-1, FtH, p-ERK, total ERK, p-JNK, and total JNK by Western blot analysis. Membranes were stripped and re-probed for GAPDH to demonstrate equal loading. **(E)** Expression of the indicated proteins in the kidneys was analyzed by densitometry, normalized to GAPDH (or total JNK or total ERK) and expressed as mean ± SEM. *p < 0.05 vs. baseline control; ^#^*p* < 0.05 vs. FtH^fl/fl^.

Intra-renal KIM-1 gene expression was rapidly induced in both genotypes and remained persistently elevated until day 7 following glycerol injection (Figure 3A). While NGAL gene expression was markedly induced in FtH^fl/fl^ kidneys after 1 day of glycerol administration, this induction was lower in myeloid FtH deficient kidneys, an effect that persisted until day 7 (Figure 3B). Intrarenal FtH mRNA transcripts were reduced early after injury in both genotypes but were restored by day 28 in both groups (Figure 3C). Concomitantly, we observed an increase in FtH protein expression at the earlier timepoints, suggesting that the decline in mRNA may correspond with enhanced translation of FtH. Corroborating our gene expression data, rhabdomyolysis was associated with an increase in intrarenal NGAL and KIM-1 protein expression (Figures 3D,E). NGAL was predominantly expressed in the tubular epithelium in a punctate pattern in the cytoplasm (Supplementary Figure 2). Induction of KIM-1 was higher in mice that were deficient for myeloid FtH at day 1 (Figure 3D). We further determined that activation of MAPK pathways, JNK and ERK, were not differentially regulated in the absence of FtH during injury (Figures 3D,E). As rhabdomyolysis is associated with excess heme burden and oxidative stress, we examined kidneys for lipid peroxidation. As shown in Figure 3D, glycerol administration is associated with an increase in lipid peroxidation in both genotypes, as evident by an increase in expression of 4-hydroxynonenal. Immunohistochemical staining of 4-HNE revealed an intense tubular expression during the acute phase of rhabdomyolysis which reduced by day 7 (Supplementary Figure 3).

### Myeloid ferritin heavy chain deficiency does not affect cell death and proliferation during rhabdomyolysis-acute kidney injury

**FIGURE 4:**
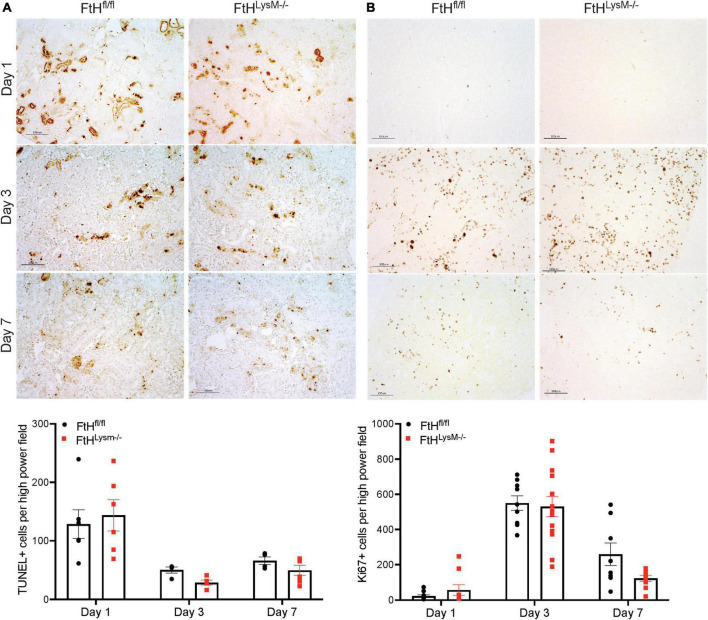
Cell death and proliferation in rhabdomyolysis-AKI. **(A)** Representative images of kidney sections in the TUNEL assay from FtH^fl/fl^ and FtH^LysM–/–^ mice after 1-, 3-, or 7-days post-glycerol administration. Brown puncta represents TUNEL positive nuclei. Scale bar represents 100 μm. **(B)** Representative images of kidney sections immunostained with Ki67 antibody from FtH^fl/fl^ and FtH^LysM–/–^ mice after 1-, 3-, or 7-days post-glycerol administration. Brown puncta represents Ki67 positive nuclei. Scale bar represents 100 μm. Quantification of TUNEL- and Ki67-positive cells, respectively, are shown in the graphs. To quantify, 5–10 high power field images were obtained, and stained nuclei were quantified, and a mean value was obtained for each animal. Group means ± SEM were then calculated and presented in the graph.

Cell death, as determined by terminal deoxynucleotidyl transferase dUTP nick end labeling (TUNEL) assay was increased at day 1 in both genotypes compared to later timepoints and was not different between the genotypes (Figure 4A). Cell proliferation, as evaluated by Ki67 immunostaining, was only found to be significantly elevated at day 3 and no significant difference between the genotypes was observed (Figure 4B). These data collectively indicated that loss of myeloid FtH did not impact early response to rhabdomyolysis-induced cell death and cell proliferation.

### Rhabdomyolysis-induced kidney fibrosis is influenced by myeloid ferritin heavy chain expression

**FIGURE 5:**
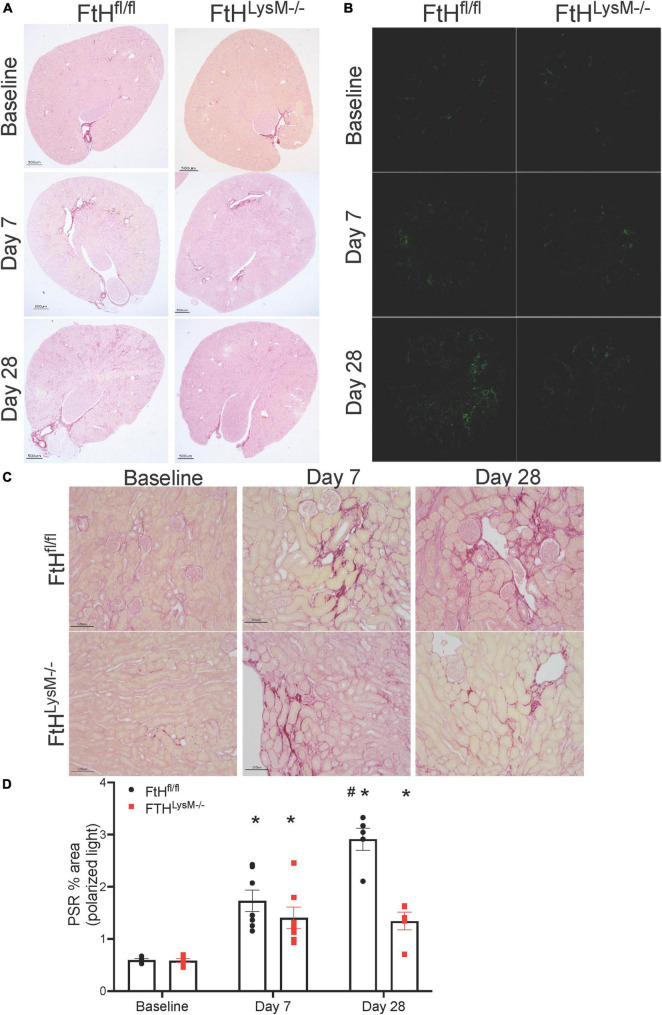
Myeloid FtH deficiency is associated with reduced fibrosis. Picrosirius staining was performed on the kidney sections to determine fibrosis following injury. **(A,B)** Representative images of the stained kidney sections are shown. Scale bar represents 500 μm in the upper panels and 100 μm in the lower panels. **(C)** Representative images of the stained kidney sections under polarized light are shown. **(D)** Collagen deposition was quantified under polarized light and data are expressed as percent area. ^#^*p* < 0.05 vs. FtH^fl/fl^.

**FIGURE 6:**
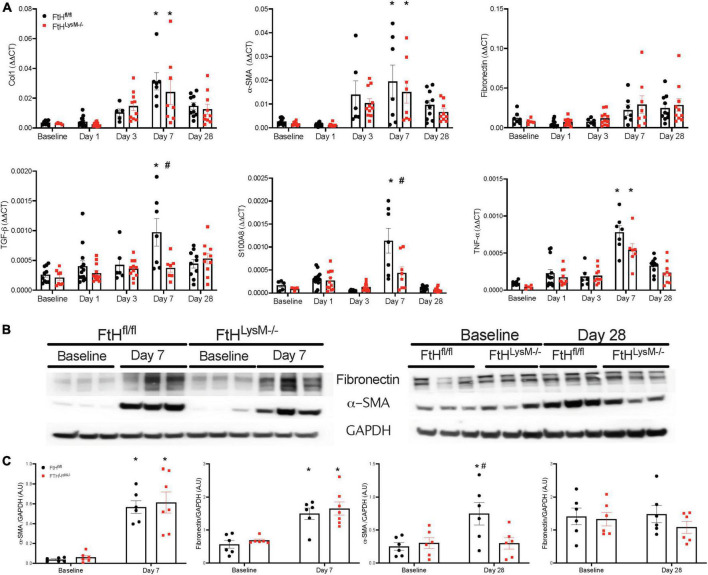
Myeloid FtH deficiency is associated with reduced expression of fibrosis markers. **(A)** Kidneys were analyzed for the expression of transforming growth factor-β, collagen 1, fibronectin, α-smooth muscle actin, S100A8, tumor necrosis factor-α by real-time PCR analysis. Each experiment was performed at least two independent times. Results were normalized to GAPDH and presented as mean ± SEM. *p < 0.05 vs. baseline control; ^#^*p* < 0.05 vs. FtH^fl/fl^. **(B)** Whole kidney lysates were analyzed for the expression of fibronectin and α-smooth muscle actin by western blot analysis. **(C)** Expression of the indicated proteins in the kidneys was analyzed by densitometry, normalized to GAPDH and expressed as mean ± SEM. *p < 0.05 vs. baseline control; ^#^*p* < 0.05 vs. FtH^fl/fl^.

While the initial damage associated with heme burden was not differentially affected by the absence of myeloid FtH, we identified that interstitial fibrosis after 4 weeks of injury was markedly lower in mice deficient for FtH. As shown in Figures 5A–D, picrosirius red staining was increased at day 7 following injury and continued to progress in FtH^fl/fl^ mice but this progression was not observed in the myeloid FtH deficient mice. Quantification of percent PSR-positive area under polarized light further confirmed these findings (Figures 5B,C). At the transcriptional level, we determined that fibrotic remodeling began as early as 3 days post-injury and peaked at 7 days, as demonstrated by increased gene expression of collagen and α-smooth muscle actin, with no significant changes in fibronectin expression (Figure 6A). At the protein level, we demonstrate notable induction of fibrotic markers at day 7 and 28 after injury (Figure 6B). Corroborating the histological data, myeloid FtH deficiency was associated with lesser induction of TGF-β at 7 days and consequently, lower expression of α-SMA at 28 days after injury (Figures 6B,C). In addition to fibrotic markers, FtH knockout mice also expressed significantly lower levels of S100A8, a potent immunomodulatory gene as well as TNF-α, a prominent pro-inflammatory cytokine during the peak of fibrogenic signaling.

### Myeloid ferritin heavy chain impacts kidney fibrosis in unilateral nephrectomy followed by rhabdomyolysis

**FIGURE 7:**
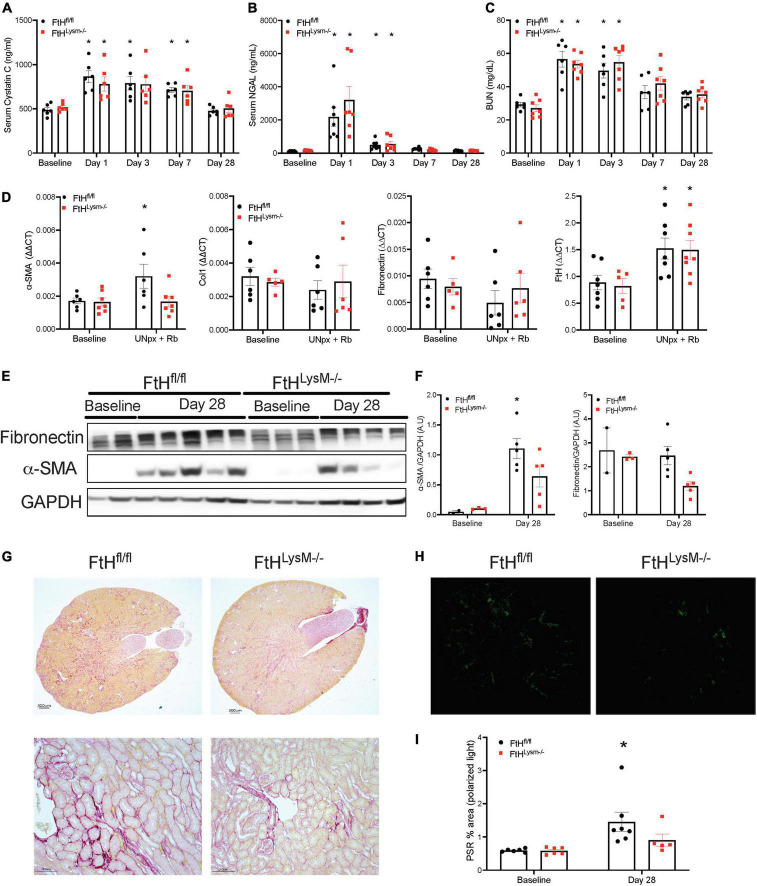
Unilateral nephrectomy followed by rhabdomyolysis leads to AKI and kidney fibrosis. Mice were subjected to unilateral nephrectomy followed by administration of 7.5 ml/kg body weight of 50% glycerol in water and harvested after 28 days. Blood was collected at various timepoints *via* facial vein and serum was analyzed for **(A)** cystatin C expressed in nanograms per milliliter (ng/ml), **(B)** neutrophil gelatinase associated lipocalin (NGAL) expressed in ng/ml, **(C)** BUN expressed in milligrams per deciliter (mg/dl). All data are expressed as mean ± SEM. *p < 0.05 vs. baseline. **(D)** Kidneys were analyzed for the expression of α-smooth muscle actin, collagen 1, fibronectin, FtH by real-time PCR analysis. Results were normalized to GAPDH and presented as mean ± SEM. *p < 0.05 vs. baseline control; ^#^*p* < 0.05 vs. FtH^fl/fl^. **(E)** Whole kidney lysates were analyzed for the expression of fibronectin and α-smooth muscle actin by western blot analysis. **(F)** Expression of the indicated proteins in the kidneys was analyzed by densitometry, normalized to GAPDH and expressed as mean ± SEM. *p < 0.05 vs. baseline control. **(G)** Representative images of the Picrosirius stained kidney sections are shown. Scale bar represents 300 and 100 μm. **(H)** Representative images of the stained kidney sections under polarized light are shown. **(I)** Collagen deposition was quantified under polarized light and data are expressed as percent area. *p < 0.05 vs. baseline.

To determine whether loss of kidney mass by unilateral nephrectomy would impact injury and fibrosis outcomes in the absence of myeloid FtH, we subjected control and myeloid FtH deficient mice to unilateral nephrectomy followed by glycerol administration. We found that glycerol induced kidney injury was similar between the genotypes, as evident by a temporal increase in levels of BUN, cystatin C, and NGAL during the early timepoints (Figures 7A–C). At 28 days following glycerol administration, expression of the fibrotic marker, α-SMA was significantly higher only in the wild-type mice (Figures 7D–F). Additionally, picrosirius red staining for fibrosis further confirmed the increase in fibrosis in the wild-type mice, an effect that was dampened in the absence of myeloid FtH (Figures 7G–I).

### Rhabdomyolysis-induced immune cell infiltration is not impacted by ferritin heavy chain

**FIGURE 8:**
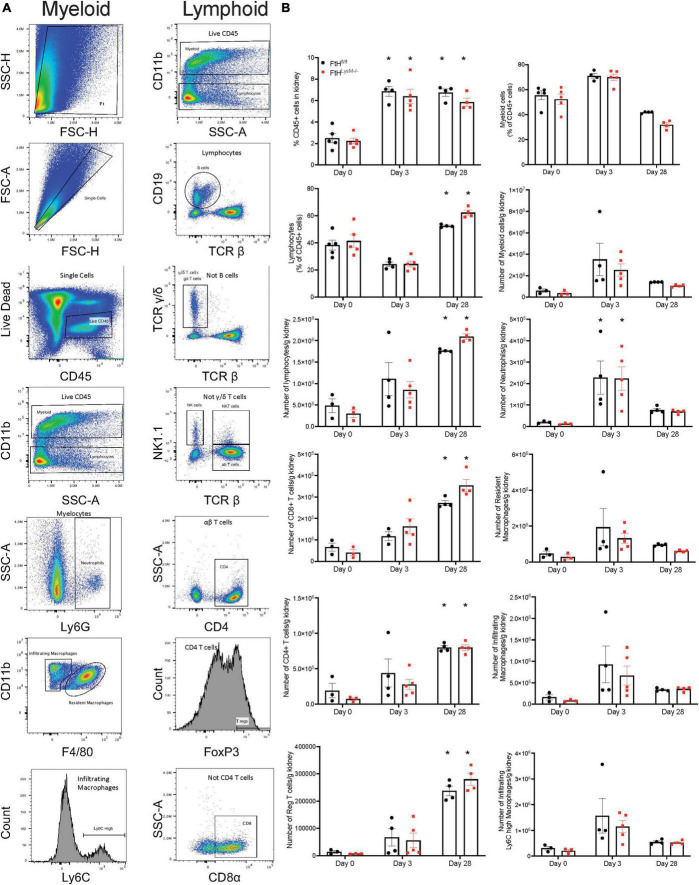
Myeloid FtH deficiency does not affect rhabdomyolysis-induced accumulation of intra-renal immune cell populations. **(A)** Representative flow cytometry histograms of kidneys demonstrating the gating scheme to identify immune cell populations. **(B)** Quantification of the immune cell populations. Data are presented as proportion of CD45+ cells or presented as number of cells per gram kidney and expressed as mean ± SEM. *p < 0.05 vs. baseline control.

To determine whether the difference observed in fibrosis could be attributed to immune cell populations, we next assessed intrarenal lymphoid and myeloid cell populations after glycerol administration (Figure 8). Relative to quiescent controls, both genotypes experienced a similar rise in percent CD45+ cells that remained elevated until day 28. We also observed a temporal response in the lymphoid and myeloid populations after injury. Specifically, myeloid cells dominated the leukocyte population at early timepoints whereas, lymphoid cells were the dominant leukocyte population at day 28. We further analyzed the myeloid cell populations and determined that loss of myeloid FtH did not impair trafficking of neutrophils and infiltrating macrophages into the kidneys during injury and also did not influence accumulation of resident macrophages. Upon further characterization of the lymphoid populations, we determined that rhabdomyolysis was associated with a temporal increase in CD4+, CD8+, and regulatory T cells, with maximal abundance identified on the day 28 after injury. Overall, difference in fibrotic remodeling in the FtH deficient mice could not be attributed to alterations in absolute numbers of intra-renal immune cell populations during rhabdomyolysis.

## Discussion

In this study, we sought to determine the impact of macrophage (myeloid) FtH expression on acute and long-term effects of rhabdomyolysis-induced AKI. We demonstrated that while the early injury response to myoglobin burden was similar, loss of FtH was associated with lesser fibrosis at later timepoints. Intriguingly, this difference in fibrosis did not translate to preserved kidney function in either model. We also report that a single rhabdomyolysis event is associated with the presence of casts and marked accumulation of lymphocytes, specifically T cells, in the kidneys.

Rhabdomyolysis is a common occurrence with varying severity ranging from transient kidney injury to death. During rhabdomyolysis, damage to the muscle results in the release of myoglobin into the circulation, which then rapidly accumulates in the kidney. Within the kidney, myoglobin is detoxified by the coordinated action of heme oxygenase and ferritin. The former enzyme catalyzes the breakdown of pro-oxidant, heme and the latter safely sequester free iron. We and others have demonstrated the salutary role of these enzymes in rhabdomyolysis-induced AKI (10–13, 15). These studies were also corroborated by the use of iron chelators and inducers of heme oxygenase expression within the kidney (9, 10, 14, 15, 37, 38). More importantly, we previously identified that proximal tubular expression of heme oxygenase and ferritin was most beneficial during AKI (13, 16). Here, we substantiated those findings and identified that myeloid FtH expression did not influence the early injury response to rhabdomyolysis. We found that kidney injury and damage, as evident by a rise in serum creatinine, BUN, KIM-1, NGAL, and cystatin C levels were increased in both genotypes and returned to baseline by 28 days following injury. These functional changes paralleled structural damage (casts and necrotic tubules) observed in the kidneys up to 7 days following muscle breakdown. Corroborating previous reports, we identified that NGAL was rapidly induced in the tubular epithelium during AKI (39, 40). We also corroborated previous reports that identified maximal expression of 4-HNE, a product of lipid peroxidation in the tubular epithelium and to a lesser extent in the glomeruli and interstitial cells (8, 24). This is in contrast with a recent report that found marked expression of 4-HNE only in the glomerulus and interstitium of hypertensive rats that underwent ischemia reperfusion injury (41). We speculate that the expression pattern is reflective of the site of maximal injury, which is predominantly tubular epithelium during rhabdomyolysis.

Previous studies identified a pathogenic role for JNK and ERK signaling pathways in rhabdomyolysis-AKI (7, 21, 42–44). FtH regulates JNK activation in hepatocytes (45). JNK activation upregulates pro-fibrotic cytokine production and promotes chronic kidney fibrosis (46). Here, we found that myeloid FtH did not impact activation of these signaling pathways in the kidney. Corroborating previous reports, we identified that lipid peroxidation and cell death were increased at 1-day post-injury (7, 47, 48). We also found that cell proliferation peaked at day 3 and returned to baseline within 7 days after glycerol administration. These changes were accompanied by a rapid infiltration of neutrophils and infiltrating macrophages at 3 days post-injury. Additionally, in animals that were subjected to unilateral nephrectomy followed by rhabdomyolysis, we found elevations in injury markers at the early timepoints that did not significantly differ between the genotypes, underscoring the tubular handling of myoglobin as the dominant effector during the acute phase. Despite the loss of kidney mass in this model, BUN and other injury markers returned to baseline by 28 days, precluding us from determining the effects of myeloid FtH on AKI to CKD transition. Taken together, these data suggest that the early injury response to the insult were not altered by myeloid FtH expression.

Few studies have examined the long-term outcome of an acute insult, especially after kidney function is restored. Given the significance of macrophages in mediating repair and fibrosis following rhabdomyolysis (22), we examined mice after 28 days of glycerol administration and found no difference in the accumulation of myeloid cell populations between the genotypes. However, we found a significant increase in lymphocytes, specifically T cells in the kidneys. We also determined that while the kidney architecture was markedly improved, several tubular casts persisted in the kidneys, suggesting sustained damage or incomplete recovery that was below the threshold for detection *via* biomarkers of kidney injury, but could potentially leave the kidneys more susceptible to a second hit. Corroborating previous reports, we identified that rhabdomyolysis led to a significant increase in interstitial fibrosis (49). However, this was notably lower in the absence of myeloid FtH. Upon further examination, we found that fibrotic remodeling was induced in both genotypes by 7 days post-injury, as indicated by a comparable increase in fibrotic gene and protein expression. However, collagen deposition continued to progress in the wild-type mice, an effect that was prevented in mice deficient for FtH. We also identified that expression of transforming growth factor-β, a pro-fibrogenic mediator, was significantly induced in wild-type mice at 7 days post-injury but this was markedly blunted in the absence of myeloid FtH. Interestingly, myeloid FtH driven fibrotic remodeling was observed in animals that were subjected to unilateral nephrectomy followed by rhabdomyolysis despite similar injury between genotypes. These findings further strengthen the role of myeloid FtH in fibrotic remodeling after injury.

Mechanistically, we identified that immunomodulatory genes, such as S100A8 and TNF-α were significantly upregulated in wild-type mice compared to FtH deficient mice at 7 days post-injury and may contribute to the pathogenic fibrosis observed at 28 days. In this context, a kidney biopsy of a patient with rhabdomyolysis-AKI had increased TNF-α and lipid peroxidation and was accompanied by infiltration of myeloid cells and lymphocytes (8). Also, transgenic mice deficient in S100A8 demonstrated reduced kidney damage and fibrosis following AKI. S100A8 is a potent inducer of TLR4 signaling and subsequently impacts kidney injury responses (50, 51). In human and rodent kidneys, S100A8 is mainly confined to the myeloid compartment during injury (50). We speculate that myeloid FtH may regulate S100A8 expression and mediate cross-talk with kidney parenchymal cells, leading to fibrosis.

While our study provided novel observations, we acknowledge its limitations. First, our study is mainly observational. Our primary objective was to study the impact of FtH expression in myeloid cells on acute and long-term consequences of rhabdomyolysis. We found significant differences in fibrosis in the absence of FtH but the mechanism involved is still unclear. Our study highlights the differential expression of S100A8 and other genes implicated in injury as potential key mediators, but further studies are warranted. Second, we characterized immune cell populations only during the injury and resolved phase after rhabdomyolysis. This precluded us from determining the role of FtH in regulating macrophage phenotype and consequently, its effect on fibrotic remodeling. Also, this current study did not employ unbiased RNA sequencing or proteomic approaches to examine pathways that are relevant in cross-talk mechanisms between immune cells and kidney parenchyma. A recent study demonstrated the role for myeloid derived arginase expression in mediating cross-talk with proximal tubules to promote repair following AKI (52). Elegant work by Belliere et al. demonstrated that macrophage phenotype impacts kidney outcomes during rhabdomyolysis (22, 24, 30). To this effect, we previously demonstrated that macrophage activation and polarization are regulated by expression of FtH (36). Third, we used a singular low dose glycerol to avoid mortality observed with higher doses of glycerol (10). While this dose allowed us to perform long-term evaluations of fibrosis, kidney function returned to pre-injury levels. This finding is in corroboration with a recent report by Menshikh et al. that demonstrated marked interstitial fibrosis after rhabdomyolysis, but kidney function was preserved at 5 weeks after injury (49). Our second model employed unilateral nephrectomy coupled with rhabdomyolysis, and recapitulated our initial observations.

Despite these limitations, we report several key findings. First, in corroboration with previous reports, we demonstrate that a single, transient insult to the kidney, while insufficient to cause prolonged loss of kidney function, may result in long-term fibrosis (22, 49). These changes, although seemingly benign, may have repercussions on the ability of the kidneys to withstand further insults. Our second model employed unilateral nephrectomy coupled with rhabdomyolysis to increase the severity of AKI. This model re-affirmed our earlier findings that showed no impact of myeloid FtH during the acute phase after rhabdomyolysis but prevented the progression of fibrosis at later timepoints. Previous studies highlighted the protective role of tubular expression of heme-iron detoxifying proteins in rhabdomyolysis (53, 54). Our study identified that expression of iron-sequestering FtH in the myeloid compartment did not influence rhabdomyolysis-induced injury. We speculate that a second insult after rhabdomyolysis-mediated fibrotic remodeling would increase the severity of AKI or progression of fibrosis. Second, we report that rhabdomyolysis is associated with a rapid accumulation of myeloid cells during the acute phase and lymphocytes during the delayed phase after injury. The implications of such immune cell infiltration are currently being investigated. Third, we found that myeloid FtH deficiency led to less fibrosis compared to wild-type mice after a single dose of glycerol administration. Additionally, mice that underwent unilateral nephrectomy followed by glycerol administration demonstrated heightened fibrotic remodeling in wild-type mice that was markedly lower in the absence of myeloid FtH. Intriguingly, we reported similar fibrotic remodeling in the absence of myeloid FtH during obstructive nephropathy (36). These findings confirm the role of myeloid FtH in regulating kidney fibrosis. This work highlights the potential for targeted macrophage therapies when mitigation of the acute injury is not possible. Future studies are warranted to determine whether the differential fibrotic remodeling will render these animals more susceptible to a second AKI insult or progress to CKD at an accelerated pace.

## Data availability statement

The raw data supporting the conclusions of this article will be made available by the authors, without undue reservation.

## Ethics statement

The animal study was reviewed and approved by the Institution Animal Care and Use Committee of the University of Alabama at Birmingham.

## Author contributions

KRM, MT, JA, and SB formulated the hypothesis, designed the study, performed most of the experiments, and wrote the manuscript. EE, KHM, and JG performed and analyzed data from the flow cytometry experiments. AZ, JA, and SB performed the Western blot analysis. AT performed the analysis on kidney fibrosis. AS and MT performed the facial bleeds and collected the serum for analysis. MH, JA, VV, and MT performed the immunohistochemical analyses. All authors provided scientific input, and read, edited, and approved the manuscript.

## Supplementary material

Supplementary Figure 1 Repeated measurements of serum creatinine following rhabdomyolysis. Mice were administered 7.5 ml/kg body weight of 50% glycerol in water and blood was collected *via* facial vein bleed at baseline, days 1, 3, 7, or 28 days. Serum was analyzed for creatinine expressed in milligrams per deciliter (mg/dl), Trend lines depict temporal changes in creatinine in each mouse over time. **p* < 0.05 vs. baseline control.

Supplementary Figure 2 Expression of NGAL in the kidneys during rhabdomyolysis-AKI. Representative images of kidney sections immune-stained with NGAL antibody from FtH^fl/fl^ and FtH^LysM–/–^ mice after 1-, 3-, or 7-days post-glycerol administration. Brown color represents positive staining. Scale bar represents 100 μm.

Supplementary Figure 3 Expression of 4-HNE in the kidneys during rhabdomyolysis-AKI. Representative images of kidney sections immune-stained with 4-HNE antibody from FtH^fl/fl^ and FtH^LysM–/–^ mice after 1-, 3-, or 7-days post-glycerol administration. Brown color represents positive staining. Scale bar represents 100 μm.
