# Supplementary figures and images for: Functional consequence of myeloid ferritin heavy chain on acute and chronic effects of rhabdomyolysis-induced kidney injury
Source: Front Med (Lausanne). 2022 Sep 8;9:894521. doi: 10.3389/fmed.2022.894521 (PMC9492979; doi:10.3389/fmed.2022.894521)

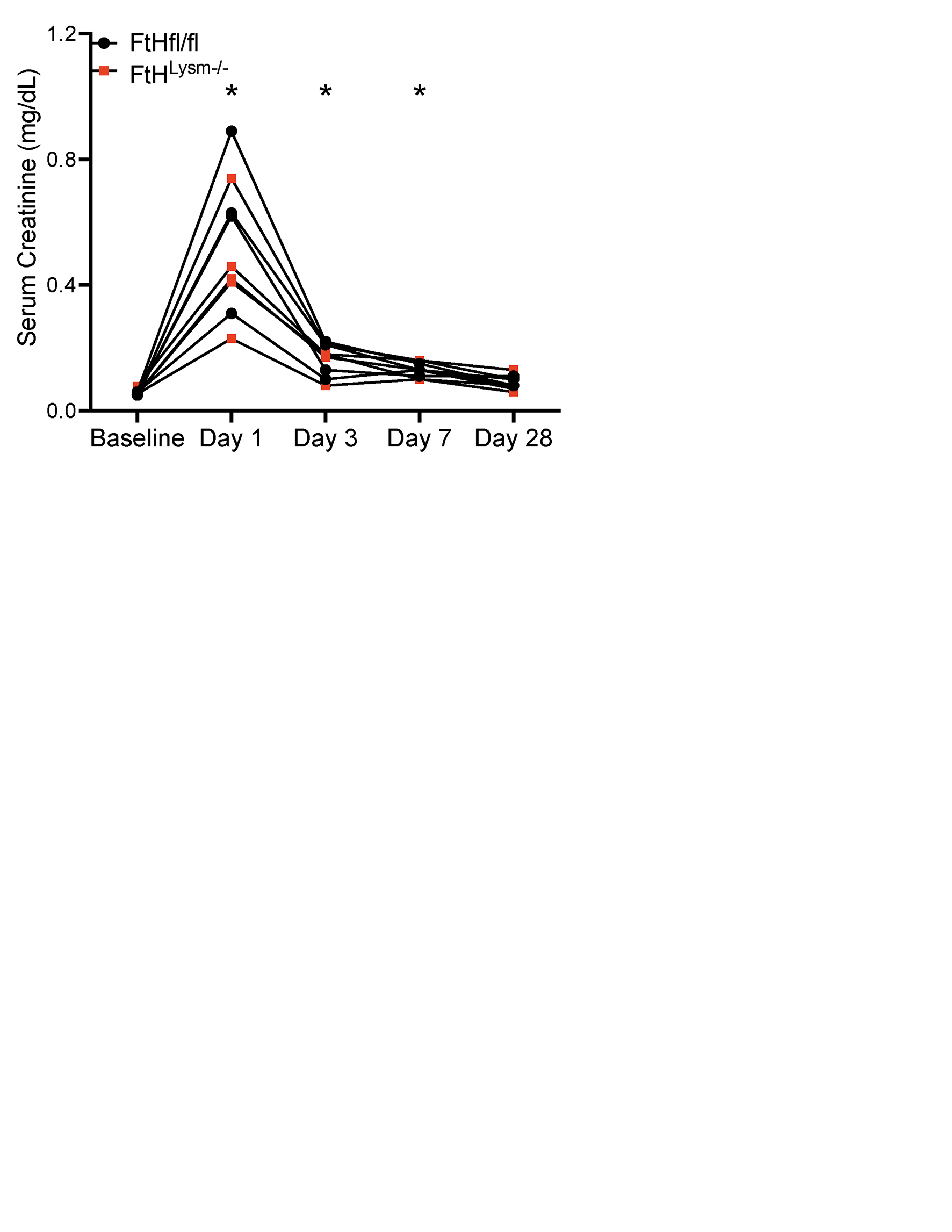

Supplement: Supplementary Figure 1 — Repeated measurements of serum creatinine following rhabdomyolysis. Mice were administered 7.5 ml/kg body weight of 50% glycerol in water and blood was collected via facial vein bleed at baseline, days 1, 3, 7, or 28 days. Serum was analyzed for creatinine expressed in milligrams per deciliter (mg/dl), Trend lines depict temporal changes in creatinine in each mouse over time. *p < 0.05 vs. baseline control. [file Image_1.TIFF]

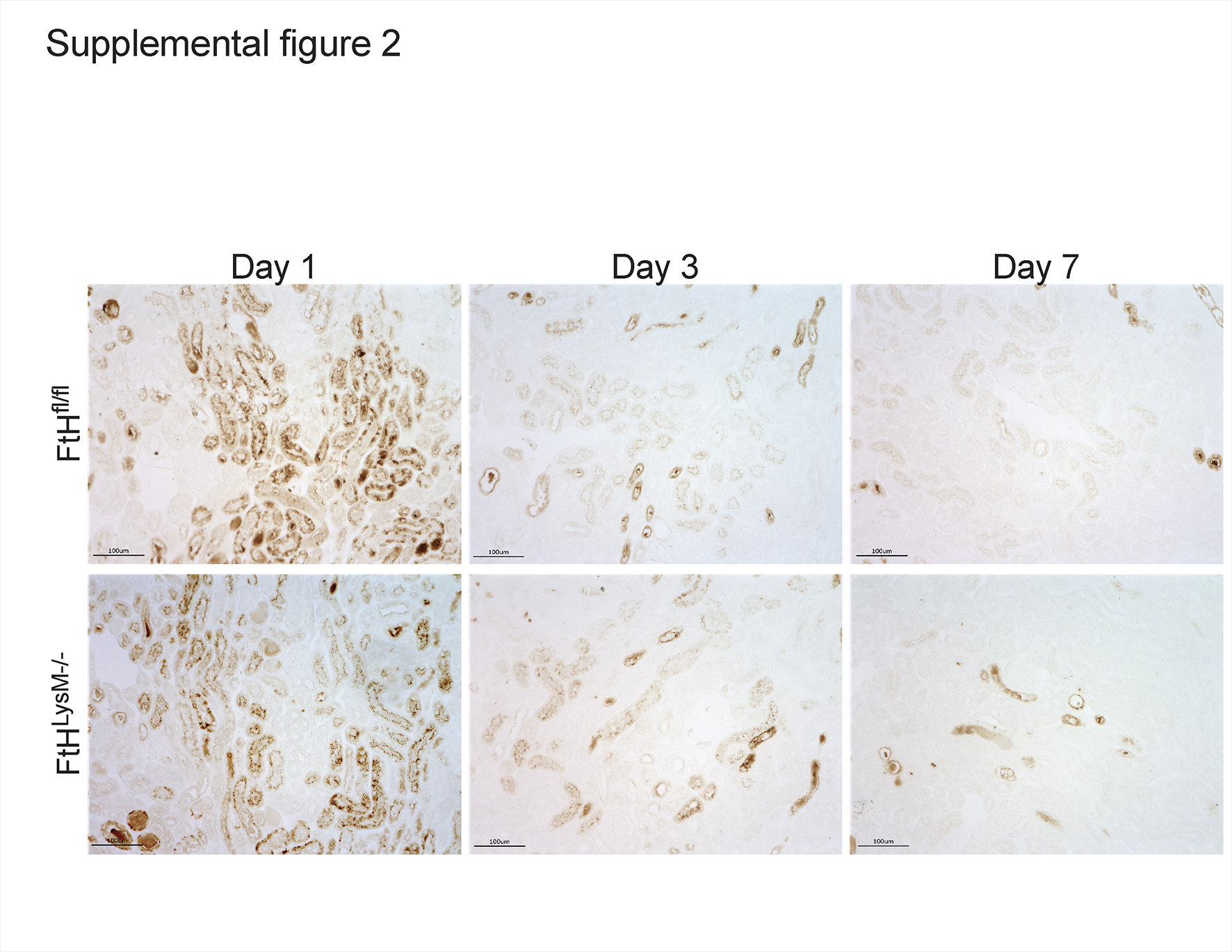

Supplement: Supplementary Figure 2 — Expression of NGAL in the kidneys during rhabdomyolysis-AKI. Representative images of kidney sections immune-stained with NGAL antibody from FtHfl/fl and FtHLysM–/– mice after 1-, 3-, or 7-days post-glycerol administration. Brown color represents positive staining. Scale bar represents 100 μm. [file Image_2.TIFF]

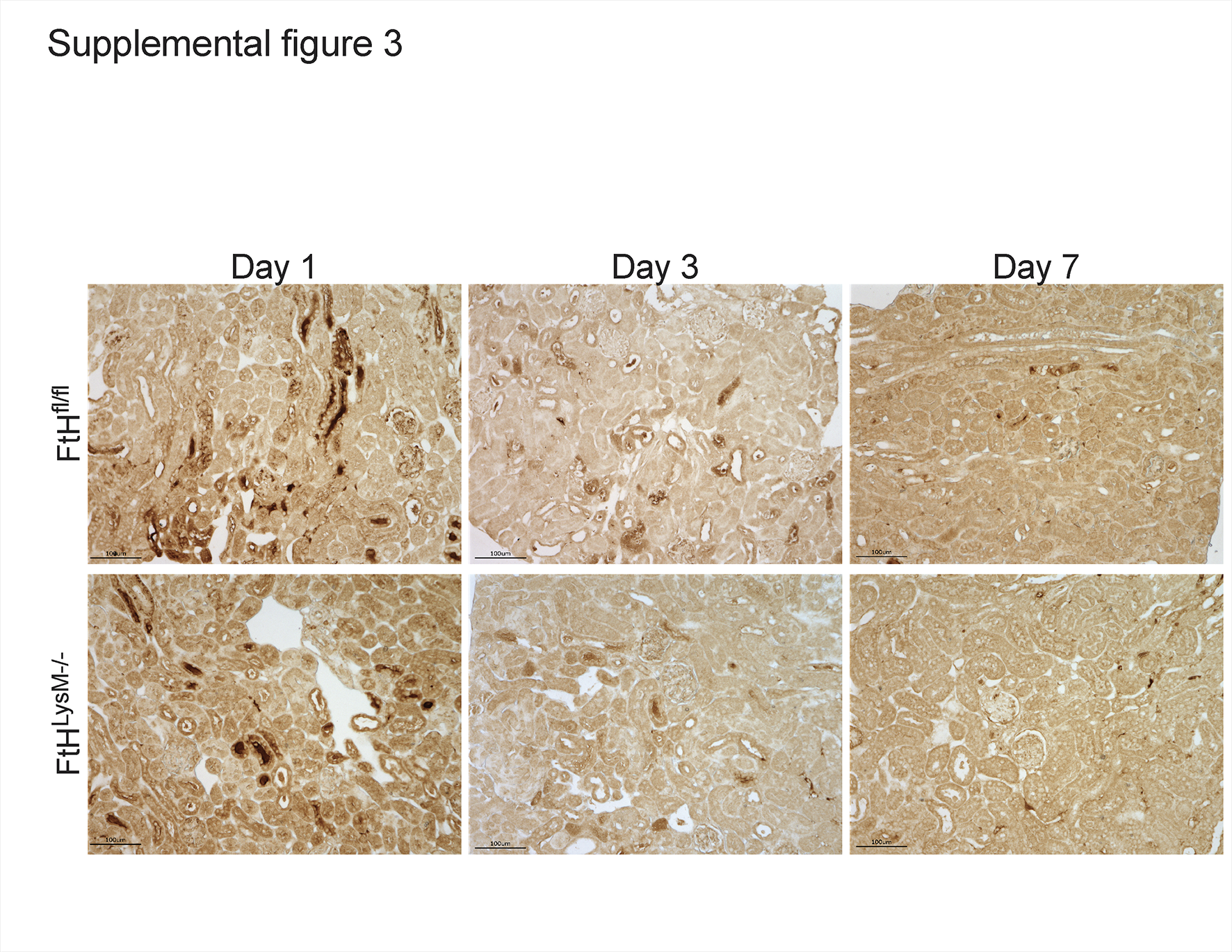

Supplement: Supplementary Figure 3 — Expression of 4-HNE in the kidneys during rhabdomyolysis-AKI. Representative images of kidney sections immune-stained with 4-HNE antibody from FtHfl/fl and FtHLysM–/– mice after 1-, 3-, or 7-days post-glycerol administration. Brown color represents positive staining. Scale bar represents 100 μm. [file Image_3.TIFF]
